# Anti-angiogenic effect of the combination of low-dose sorafenib and EGCG in HCC-induced Wistar rats

**DOI:** 10.12688/f1000research.109142.3

**Published:** 2022-12-21

**Authors:** Andry Irawan, Erik Prabowo, Ignatius Riwanto, Wahyuni Lukita Atmodjo

**Affiliations:** 1 Department of Digestive Surgery, Diponegoro University, Semarang, Central Java, Indonesia; 2 Department of Anatomy, Pelita Harapan University, Tangerang, Banten, Indonesia

**Keywords:** Sorafenib, Epigallo-3-Catechin Gallate, Vascular Endothelial Growth Factor, Microvascular Density, N-Nitrosodiethylamine

## Abstract

**Background:**

Sorafenib is a standard drug used for advanced hepatocellular carcinoma but is often resistant and toxic. Its combination with epigallo-3-catechin gallate leads to reduced resistance and toxicity but an equally effective anti-angiogenic effect.Therefore, this study aims to assess the anti-angiogenic effect of standard-dose Sorafenib compared to the combination of low-dose Sorafenib and epigallo-3-catechin gallate.

**Methods:**

We conducted an animal study and double-blind, randomized controlled trials. A total of 25 male Wistar rats (7 weeks old) were randomly divided into four groups, namely Sham (K), Control (O), a combination of low-dose Sorafenib and epigallo-3-catechin gallate group (X1), and standard-dose Sorafenib group (X2). All groups were injected with N-Nitrosodiethylamine 70 mg/kg body weight (BW) intraperitoneally for ten weeks, except the Sham group. After the development of hepatocellular carcinoma, X1 and X2 were treated for two weeks. Subsequently, liver tissues and tumor masses were examined for vascular endothelial growth factor (VEGF) level and microvascular density expression.

Results:

There was a significant difference (p=0.007) in the level of VEGF between group X1 (low dose Sorafenib + EGCG) and X2 (Standard dose Sorafenib). However, the differences in VEGF levels of group X1 and X2 compared to group O(Control) were significantly lower, with values p=0.000136 and p=0.019, respectively. The expression of microvascular density between groups X1 and X2  was not entirely different. Meanwhile, a significant difference (p<0.05) was discovered when both groups were compared with the control group.

Conclusion:

The combination of low-dose Sorafenib with epigallo-3-catechin gallate is superior in reducing the level of VEGF compared to standard-dose Sorafenib and is better than the control. Standard-dose Sorafenib and the combination of low-dose Sorafenib and epigallo-3-catechin gallate have similar effectivity in reducing the expression of microvascular density and could prevent resistance and lower toxicity effects.

## Introduction

Hepatocellular carcinoma (HCC) is the most common primary type of liver cancer. In 2013, the prevalence of liver and bile duct cancer in a developed country like the United States was 30,640. ^1^^,^^2^ A high incidence of HCC was discovered in South and East Asia, Central and West Africa, Melanesia, and Micronesia/Polynesia. It has been estimated that there are more than 749,000 new cases of HCC in men and 226,000 in women every year. ^3^^,^^4^ In 2020, liver cancer was considered the sixth most common cancer and the third leading to cancer-related death worldwide. ^5^

Vascular endothelial growth factors (VEGF) are essential in HCC tumor growth. Several carcinogens and tumor promoters initiate inappropriate activation of nuclear factor kappa B (NF-kB), which mediates the inflammation process and tumorigenesis. Meanwhile, overexpression of VEGF increases blood vessel permeability, leading to the differences between oxygen flow and delivery. A high level of VEGF is also typical in chronic liver disease that often triggers HCC. ^6^^,^^7^ Micro-vessel density (MVD) is a tumor indicator of angiogenesis that needs to be examined in HCC since a higher level of MVD shows a poor prognosis. This high angiogenic activity can be inhibited through the administration of anti-angiogenic drugs. ^8^

The first line management of HCC for operable cancers is liver resection, while chemotherapy and targeted therapy are required in an advanced cases (unresectable tumors). It was discovered that 80% of HCC patients are diagnosed with advanced-stage or inoperable cancer. Systemic treatment with Sorafenib is required to change the condition at the operable stage. Sorafenib has been proven to be the first systemic therapy that successfully improved HCC patients’ survival rate. It is an oral multi-kinase inhibitor that targets vascular endothelial growth factor receptor (VEGFR)-1, VEGFR-2, and VEGFR-3 thereby reducing tumor angiogenesis. The disadvantages of Sorafenib treatment include high cost, and approximately 30% of all patients responded to the treatment. Monotherapy of Sorafenib can cause several patient complaints, resistance, and increased charges; therefore, when given at a low dose and in combination with herbal medicines, the same effect is expected, which is more affordable in price. ^9^^–^^12^

Epigallocatechin-3-gallate (EGCG) from Sigma-Aldrich is an active ingredient that was proven to prevent the growth of blood vessels in experimental animals. Its mechanism of action is by inhibiting urokinase and tyrosine kinase, which activates VEGF, epidermal growth factor (EGF), and fibroblast growth factor (FGF). ^13^ In 2005, a previous study in Japan stated that EGCG induces both *in vitro* and *in vivo* liver cell apoptosis to improve the prognosis of HCC. *In vitro* studies showed that the effective level of EGCG varies from 1 to 100 mol/L. According to pre-clinical studies in rats, less than 5% of oral catechin taken as a tea constituent can reach systemic circulation; therefore, intraperitoneally administration is considered more effective. EGCG is the right choice to be combined with Sorafenib in advanced HCC, which uses the synergism of the two drugs. This combination can lead to similar effects as the Sorafenib standard dose. ^14^^,^^15^

Therefore, this study investigates the effectiveness of anti-angiogenic activity between Sorafenib standard dose and the low dosage of Sorafenib with EGCG.

## Methods

### Induction of HCC in animals and experimental design

**Figure 1.**
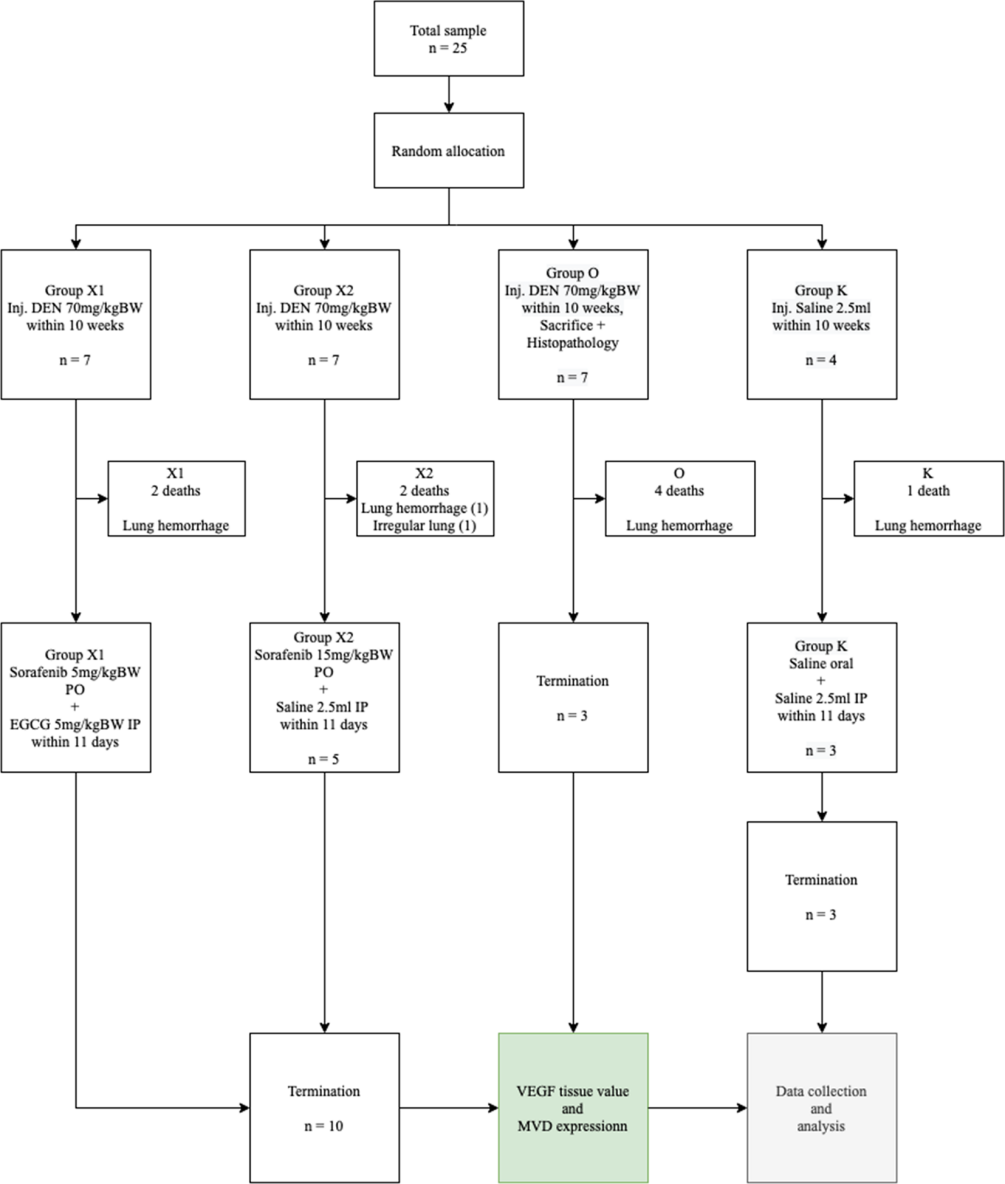
Consolidated report. DEN: Diethyl-Nitrosamine, EGCG: Epigallocatechin-3-gallate, MVD: Micro-vessel density, VEGF: Vascular endothelial growth factor.

This study used a randomized, double-blind control trial post-test only design method performed by laboratory analysts ( Figure 1). Preparation of Wistar rats began with acclimatization for three weeks in the Mochtar Riadi Institute of Nanotechnology animal laboratory. A total of 25 male Wistar rats (PT Biomedical Technology Indonesia), seven weeks old with bodyweight 200–250 grams, were placed in a cage with a controlled temperature of 22°C under 12 hours of light and dark cycle. The rats were given free access to food with AIN76 standard dietary formula for rodents, which was 67.7% carbohydrates, 11.5% lipids, and 20.8% protein from the Food Engineering Laboratory, IPB, Bogor, Indonesia, purchased from PT Surya Science and Beverages.

Diethyl-Nitrosamine (DEN) (N0756) with a molecular weight of 102.14 and Epigallocathecin-3-O-Gallate (Y0001936; primary pharmaceutical grade standard) with a molecular weight of 458.37 was purchased from Sigma-Aldrich. In contrast, each tablet of Sorafenib contains 200 mg of Tosylate (Nexavar). The DNA-carcinogen complexes resulted from the interaction between DEN and DNA through the formation of the covalent bonds. Then, DEN will induce chronic inflammation and fibrosis. The DEN-induced rat model was believed to contribute to immune response and tumor microenvironment in hepatocarcinogenesis. The characterization of anti-tumor adaptive immune response and the role of T and B cells was used to control tumor formation and progression. Under a microscope, a solid growth pattern with anaplastic cells, pleomorphic, dense chromatin, and nucleolus prominent with invasive growth into stroma was seen as similar to HCC. Consequently, genotoxic carcinogens are the most frequently used to induce HCC in Wistar rats. ^16^^–^^20^

This study followed the National Institutes of Health Guidelines for the Care and Use of Laboratory Animals. It was approved by Mochtar Riady Institute for Nanotechnology Ethics Committee (MRIN EC) with protocol number 2101001-AS06. The inclusion criteria were healthy and active 7-week-old male Wistar rats weighing 200–250 grams, while unhealthy male Wistar rats with anatomical anomalies were excluded. Any infected or dead Wistar rats were also dropped out during the experiment. We chose Wistar rats due to their short lifespan and breeding capacity. On the other hand, the metabolizing pathways induced by DEN in Wistar rats were similar to humans. Thus, this model was important in our research. ^21^^,^^22^

The sample size was calculated using the degree of freedom (Minimum and Maximum sample) formula. Rats were randomized and allocated into four groups, consisting of 7 rats, except the control (K) group, which contained four rats, with a minimal sample size of three. Subsequently, DEN was injected intraperitoneally in the abdominal area below the umbilicus on 21 rats for two treatment groups and a control group, with 70 mg/kg BW/week for ten weeks. ^23^^,^^24^ After ten weeks, all rats were randomly divided into four groups, namely sham (K), Sorafenib 5 mg/kg BW + EGCG 5 mg/kg BW (X1), Sorafenib 15 mg/kg BW (X2), and without treatment (Group O). Group K was injected with saline for ten weeks, parallel with other groups. After the administration of DEN, group O was sacrificed, and a pathologist from Dr. Mintoharjo Naval Hospital performed a histopathological examination of liver tissue to show the formation of HCC. Anaplastic cells, oval nuclei, pleomorphic, coarse chromatin, and nucleolus invasive growth into stroma were observed during the examination, which confirmed HCC. The success of the induction process was determined within ten weeks.

**Figure 2.**
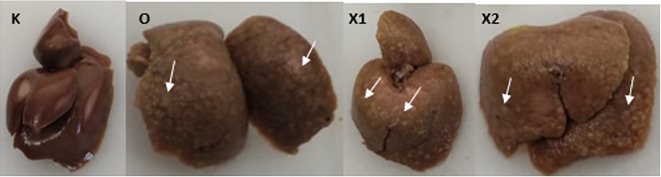
After ten weeks of Diethyl-Nitrosamine (DEN) 70 mg/kg BW intraperitoneal injection, macroscopic liver tissue. Group X1, X2, and O present multiple white nodules (White arrow); group (K) did not develop any nodules.

Sorafenib was dissolved in a maximum of 1.5 mL saline (maximum 10 mL/kg BW/day) and administered orally at 5 mg/kg BW and 15 mg/kg BW. Subsequently, EGCG 5 mg/kg BW/day was dissolved in approximately 1.5 mL saline (maximum 20 mL/kg BW/day) and administered by intraperitoneal injection once a day for 14 days. The sham group (K) was administered a saline solution orally and intraperitoneally, while the sorafenib-only group (X2) received intraperitoneal saline and oral Sorafenib. Meanwhile, the combination group of EGCG and Sorafenib (X1) received intraperitoneal EGCG and oral Sorafenib, and the bodyweight of the rats was measured once a week. At the end of the experiment, the rats were sacrificed, and exsanguination was done on deeply anesthetized animals with ketamine 80 mg/kg BW and xylazine 100 mg/kg BW intramuscularly to alleviate any suffering. The liver tissues were resected and examined microscopically. Moreover, a veterinarian performed a necropsy when any rat died during the experiment to investigate the cause of death ( Figure 2).

In liver tissue, VEGF was evaluated using an enzyme-linked immunosorbent assay (ELISA) quantitative methods, while MVD was calculated using Immunohistochemistry (IHC). The intensity and area of sinusoidal endothelial staining were measured quantitatively using a microscope at 100× magnification. Furthermore, the hot spots from the immunohistochemistry were selected using the “color selection” function and the “area/density (intensity)” function (ImageJ, RRID:SCR_003070) to calculate the level.

### ELISA for tissue VEGF

To prepare lysate from tissue, tissue of interest was dissected with clean tools. Dissected tissue was placed in microcentrifuge or Eppendorf tubes. Lysis buffer consisting of NP-40 buffer, sodium chloride, NP-40, Tris pH 8.0, and Triton X-100 or NP-40) was added to 5 mg of tissue and homogenized rapidly. Next, it was centrifuged at 4°C for 20 minutes. After carefully removing the tubes and placing them on ice, any supernatant was aspirated, and the pellet was discarded. ^25^^,^^26^

A Bradford, a Lowry, or a bicinchoninic acid (BCA) assay was conducted to calculate protein level. Bovine Serum Albumin (BSA) is usually used as a standard protein. Each sample was frozen at -20°C for immunoprecipitation. 200μL of 1X Bradford reagent, five μL of BSA, and 30 μL of the unknown model were added to each test tube. Absorbance was determined using a sipper or individual cuvettes at 595 (VIS lamp).

All standards and samples were prepared twice as recommended and stored at room temperature. Each well-containing standard and sample were incubated for 2.5 hours at room temperature. The washing process using 300 μL of Wash Buffer was repeated four times. All liquid was eliminated after each step to achieve the best result. After the last wash, the plate was inverted and blotted using a paper towel.

Approximately 100 μL of 1× Detection Antibody was titrated and incubated at room temperature for one hour. All liquid was removed, and 100 μL of Streptavidin solution was added and incubated at room temperature for 45 minutes. Approximately 100 μL of TMB One-Step Substrate Reagent (Item H) was added and set in dark condition for 30 minutes. Lastly, 50 μL of Stop Solution (Item I) was added, and absorbance was recorded at 450 nm.

### Immunohistochemistry for MVD

Deparaffinization was done in the incubator at 60°C for 45 minutes, followed by deparaffinization in xylene for 10 minutes. Next, 96% ethanol, 80% ethanol, and 70% ethanol were added to the formalin-fixed paraffin-embedded tissue for 5 minutes. The tissue was washed using distilled water. Antigen retrieval buffer (citrate buffer + tween) was placed into a jar and microwaved at full power for 20 minutes. The pot was removed and chilled on ice for 20 minutes.

Several drops of Hydrogen Peroxide Block were added to the section, incubated for 10 minutes, and rinsed in buffer twice. Protein Block was added, set for 10 minutes, and rinsed once in the buffer. Primary MVD polyclonal antibody (MBS 2520154) from Abcam was added (1:100) in PBS-T, incubated at 4°C for 2 hours, and rinsed four times in buffer. A Biotinylated Goat Anti-Polyvalent was added, set for 10 minutes, and rinsed four times in buffer. Streptavidin Peroxidase was added, incubated for 10 minutes, and rinsed four times in buffer.

Approximately 30 μL of DAB Chromogen was applied into 1.5 mL of DAB Substrate. It was incubated for 3 seconds and rinsed four times in the buffer. Next, Hematoxylin was used as a counterstain, incubated for 20 minutes, and rinsed in tap water. Tissues were dehydrated using 70% ethanol, 80% ethanol, and 96% ethanol, each for 1 minute. The samples were observed under 10×, 40×, and 100× magnification. ^27^ For MVD, the Spearman’s correlation coefficient (rho) was 0.93 (p < 0.01), while intra-observer agreement (Kappa) were 0.88 for cut-off using mean. ^28^^,^^29^

### Statistical analysis

All data were expressed as mean ± standard deviation of the mean. The statistical analysis was conducted using SPSS 28 (IBM SPSS Statistics, RRID:SCR_019096). All data were normally distributed, and the comparisons between groups were analyzed using ANOVA. *Post hoc* analysis using the least significant difference (LSD), where a p-value < 0.05 was considered statistically significant.

## Results

This study showed that the Sorafenib-only group effectively reduced VEGF tissue levels better than the treatment group. However, there was no significant difference in lowering MVD expression compared to the low-dose Sorafenib and EGCG group, which indicated better overall results than the Sorafenib-only group. During the experiment, nine rats died due to pulmonary hemorrhage, and one died of irregular lung surface.

A total of 13 rats survived the 11 weeks of the experiment, although some looked unhealthy. One group reached the minimum sample size based on the calculation of the degree of freedom, the Institutional Animal Care and Use Committee (IACUC) Guidebook, and the World Health Organization (WHO). At the end of the experiment, the whole group of mice was terminated according to the euthanasia techniques based on IACUC and the American Veterinary Medical Association (AVMA) Guidelines.

### Data characteristic

The Shapiro-Wilk test results were used to calculate the mean and the distribution of the rats’ body weight data, and p > 0.05 was obtained for all groups. Homogeneity test results with Levene’s test obtained p = 0.978, which showed that the data obtained is homogeneous.

**Figure 3.**
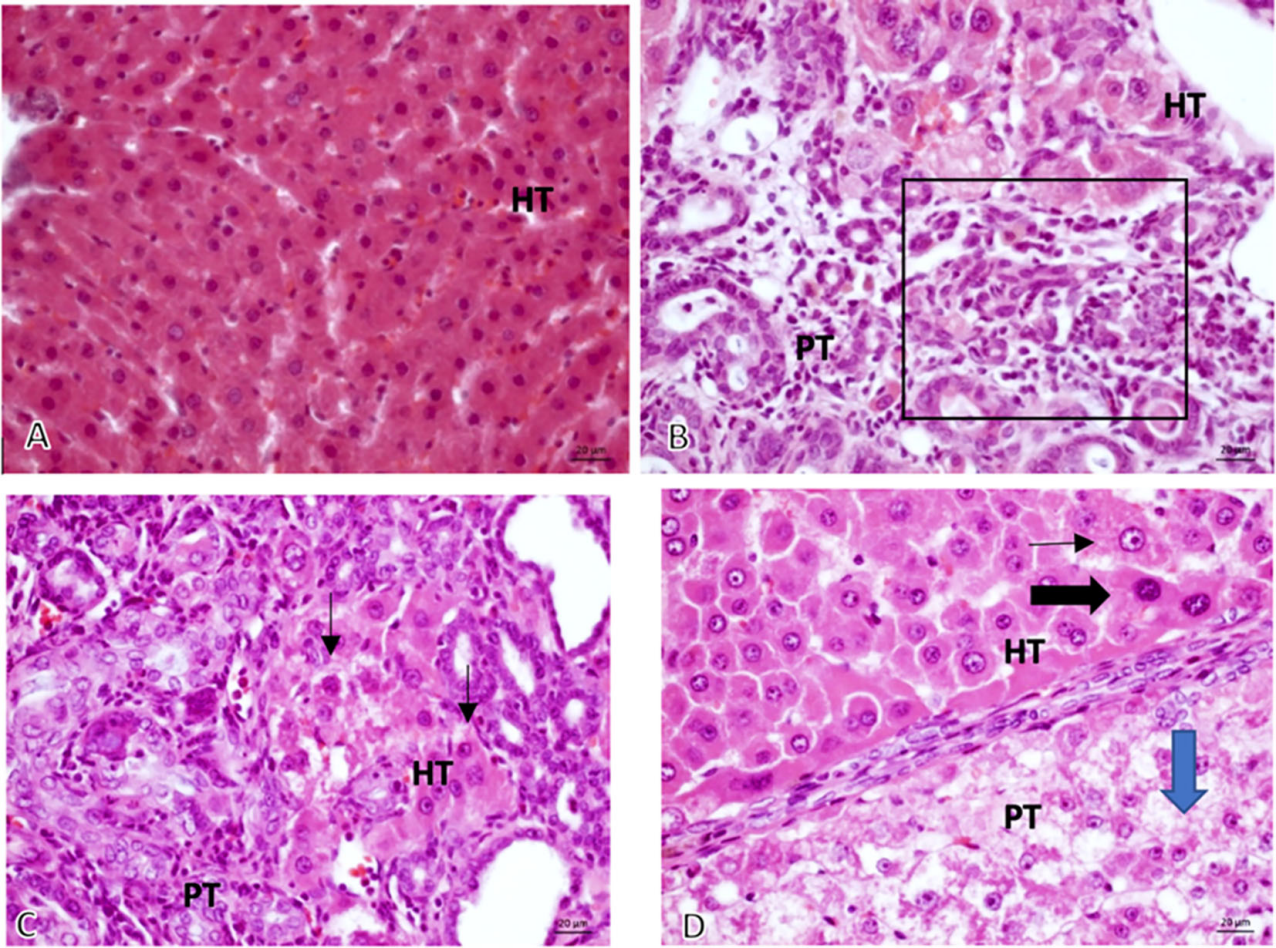
The validation of HCC. Microscopic 40× magnification on non-induced Diethyl-Nitrosamine (DEN) group: (A, Group K) normal hepatocyte; comparing graphic on DEN induced group: (B, Group O) Bile Duct hyperplasia (Black square area); (C, Group X1) Hyperchromatic cells (black arrow) and (D, Group X2) Prominent cell (thin-arrow), Hyperchromatic cell (thick black – arrow), Ballooning degeneration (Blue-Arrow). *HT, Hepatocyte; PT, Porta tract.*

The 16 sample slides revealed that the tumor growth was solid, with anaplastic cells having round, oval, pleomorphic nuclei, coarse chromatin, and prominent nucleolus that grows invasively into the stroma ( Figure 3).

### VEGF level

**Figure 4.**
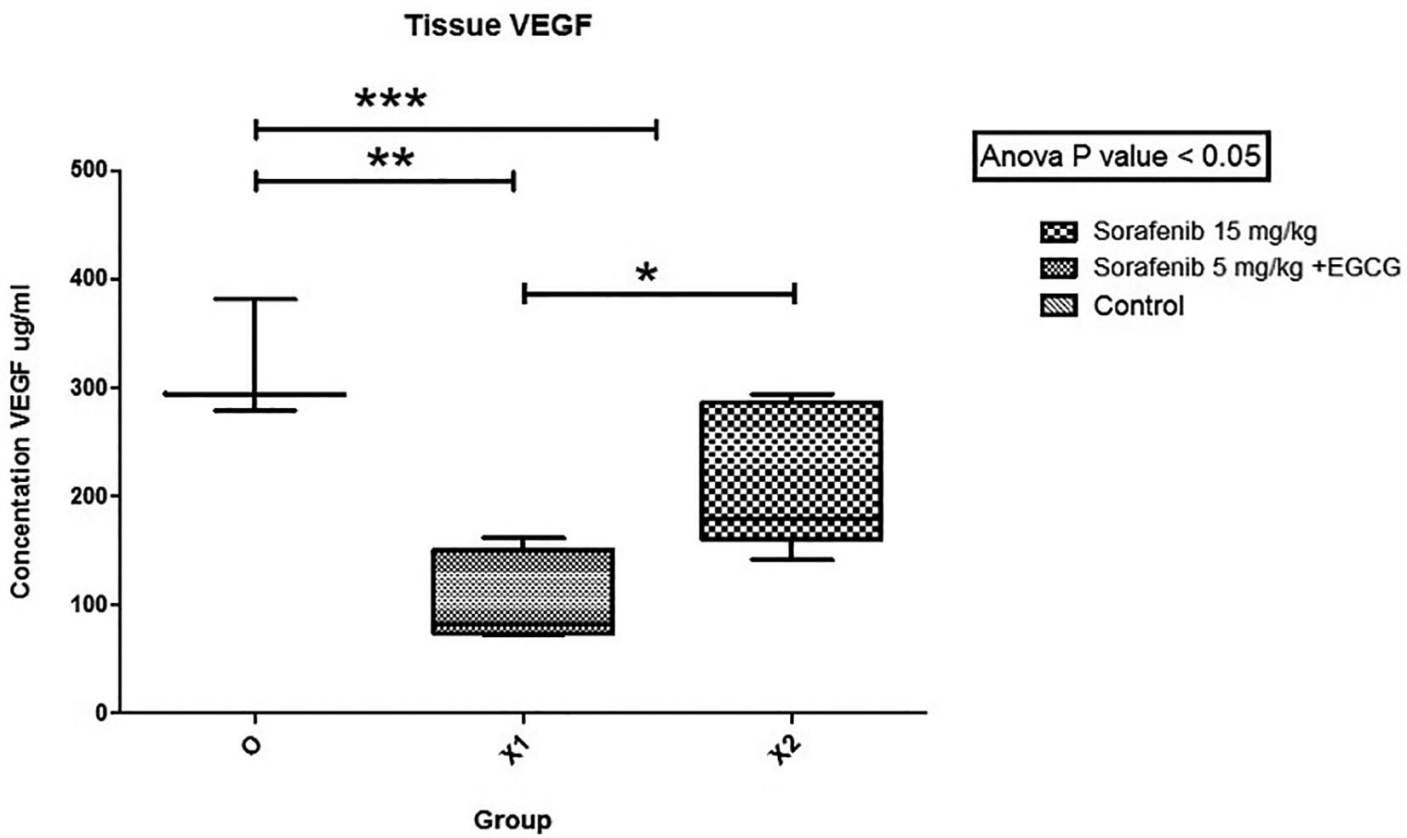
The tissue VEGF level. There was a significant difference in the level of VEGF in group X1 compared to X2 (*). Between groups X1 and O, VEGF was significantly different (**). Between-group X2 and O, VEGF was significantly different (***) (p < 0.05).

The mean VEGF level ( Figure 4) between group Sorafenib 5 mg/kg BW + EGCG 5 mg/kg BW (X1) (106.682 ± 41.024) and group Sorafenib 15 mg/kg BW (X2) (214.5162 ± 67.717) had significant difference (p < 0.05), which showed that group X1 had the most potent effect in reducing VEGF level. Furthermore, the VEGF levels between group X1 and the group without treatment (O) (318.101 ± 55.078) were significantly different (p < 0.05). A similar result was seen since the VEGF level between groups X2 and O were quite different (p < 0.05).

### MVD expression

**Figure 5.**
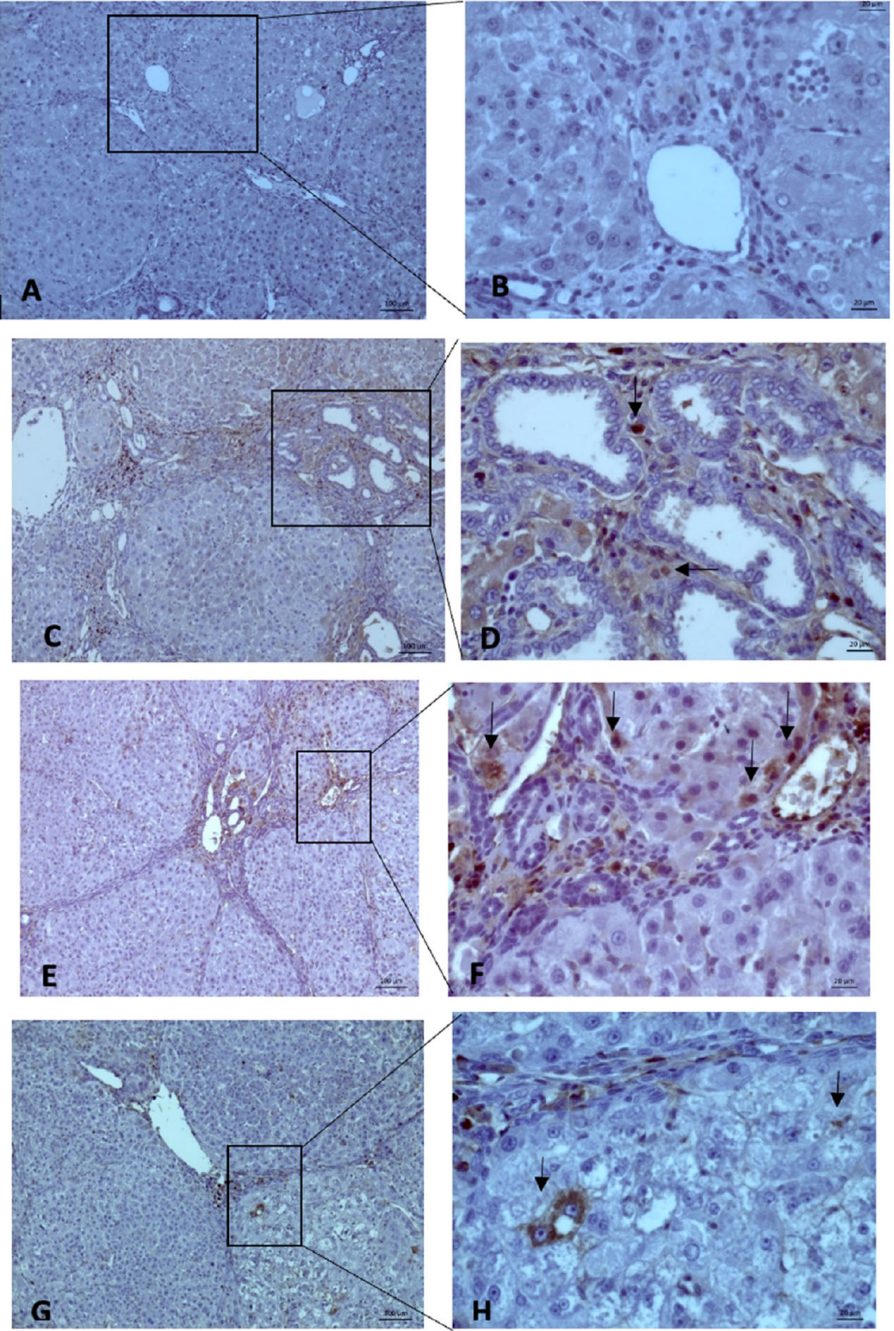
The MVD expression on microscope 10× (left side) and 40× magnification (right side): (A-B) (Group Sham) no finding of “Hot Spot” area on hepatocyte endothelial sinusoid; (C-D) (Group O) showed brown spot (black-arrow); (E-F) (Group X1) and (G-H) (Group X2) we found the same result (Black-Arrow). All three groups showed a “hot spot” area, which means there were positive results.

**Figure 6.**
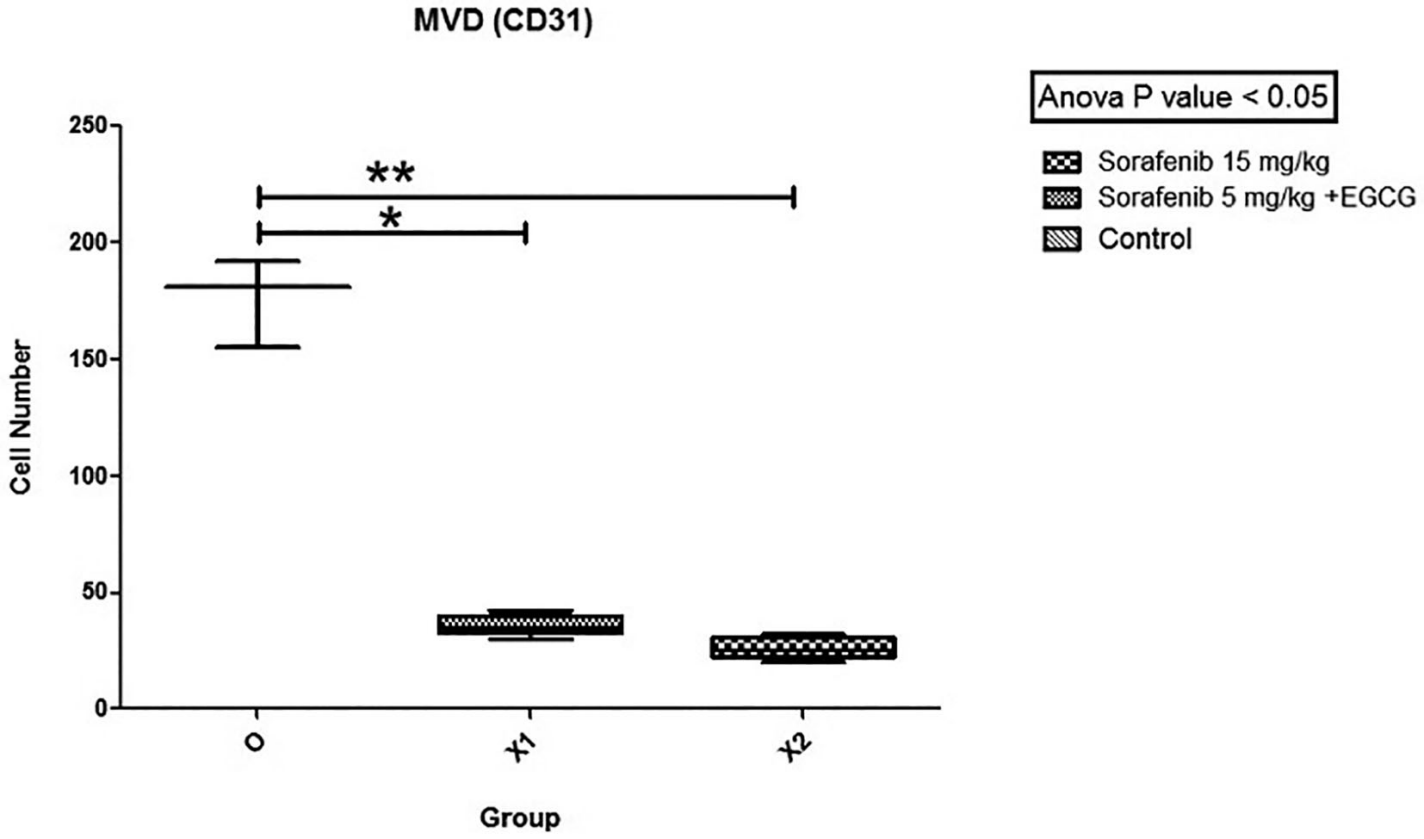
The MVD expression. There was no significant difference in the expression of MVD between group X1 and X2, but both X1 and X2 were significantly different compared to the control group (O) (non-treatment group) with (p < 0.05).

MVD evaluation was performed using 10× and 40× magnification to measure the intensity and area of sinusoidal endothelial staining ( Figure 5). Subsequently, the hot spots from the immunohistochemistry were selected, and levels were calculated. The mean MVD expression between the group Sorafenib 5 mg/kg BW + EGCG 5 mg/kg BW (X1) (36 ± 4.416) and group Sorafenib 15 mg/kg BW (X2) (26.2 ± 4.55) had no significant difference. Based on the results, MVD expression ( Figure 6) between all groups and group O (176 ± 19) showed a significant difference (p < 0.05).

## Discussion

This study showed that the benefits of the combination of Sorafenib and EGCG are the same as an anti-neoplastic drug, which is as effective as anti-angiogenic. The results of VEGF levels between 2 groups, namely the combination group Sorafenib 5 mg/kg BW and EGCG 5 mg/kg BW (X1) compared with Sorafenib 15 mg/kg BW alone (X2), showed that both treatments could reduce VEGF level. However, the X1 group was significantly more potent in decreasing VEGF value than group X2. This has exceeded the expectations, where the combination of low-dose Sorafenib and EGCG was more effective than only standard-dose Sorafenib. This indicated that Sorafenib and EGCG act synergistically with a strengthening effect in anti-angiogenesis.

*In vivo* and *in vitro* studies have proved the effect of EGCG as a chemo-preventive, anti-angiogenic, anti-invasive, anti-proliferative, anti-inflammatory, and antioxidant substance. It was shown that EGCG blocks NF-kB activation by inhibiting IκBα degradation and the mitogen-activated protein kinase (MAPK) pathway. Meanwhile, downregulation of inducible nitric oxide synthase (iNOS) transcription and nitric oxide (NO) production from macrophages depends on NF-kB inhibition. It was reported that EGCG blocks NF-kB activation in human endothelial cells and inhibits monocyte chemotactic protein-1 (MCP-1) expression. Similarly, EGCG also prevents the apoptosis process by reducing mRNA expression of Bax and caspase three activity. It also inhibits cyclooxygenase-2 (COX-2) expression, proteasome-dependent degradation, MAPK pathways, and growth factor-dependent signaling, namely insulin-like growth factor-I (IGF-I), VEGF, and EGF. ^29^

The results also suggested that several factors are responsible for the less effective administration of Sorafenib as a single drug. Firstly, Sorafenib is accumulated in cancer cells, followed by an increase in the expression of enzymes to metabolize Sorafenib, which affects drug exposure. Thirdly, the presence or absence of tumor influences the level of Sorafenib and its primary metabolites based on assessing resistance to Sorafenib administration. ^30^

MVD expression was significantly different between X1 and X2 groups compared to group O. This showed that the combination of low-dose Sorafenib and EGCG is also effective as standard-dose Sorafenib-only by decreasing MVD expression intratumorally. However, there is no significant difference in the discovery of MVD expression between the X1 and X2 groups. These are influenced by time length because the formation of MVD is affected by the growth of the capsule in the tumor. This is in line with Kuczynski EA *et al.*, who showed that therapy with Sorafenib significantly inhibits MVD (p < 0.001 vs. controls). In contrast, the Sorafenib-resistant group showed no evidence of continued angiogenesis. ^31^ The evaluation of MVD expression is critical to determine the prognosis. According to Poon RTP *et al.,* MVD-CD34 tumors were the only significant predictor of disease-free survival in patients with HCC or tumor size < 5 cm. ^32^

Although the result differed from the hypothesis, a very satisfying conclusion was successfully obtained. The combination of low-dose Sorafenib with EGCG had better effectiveness than the standard dose of Sorafenib in lowering VEGF levels and was equally effective in reducing MVD expression in Wistar rats induced by DEN. Based on the results, it was concluded that EGCG adds a supplementary anti-angiogenic effect for HCC. Therefore, using low-dose Sorafenib combined with EGCG is a more cost-effective therapy recommended to increase drug compliance potentially. Similarly, it also provides a satisfying therapeutic effect for advanced HCC.

After ten weeks of administering DEN 70 mg/kg, macroscopic gross liver tissues showed irregular surfaces and pale colors. The histopathologists also confirmed HCC characteristics. The length and dosage of DEN induction were in line with Atmodjo *et al.*, while the liver carcinogenesis or the beginning of HCC was recorded. ^33^ There was a force majeure event in the experimental animal since nine rats were found dead. Meanwhile, eight rats died from pulmonary hemorrhage, and one died with irregular lung surface due to lung injury. The hypothesis of this study stated that the suppression of the immune system increased the probability of lung disorders due to respiratory infections, fibrosis, or early malignancy in the lungs when rats were injected with DEN. ^34^ This is because one rat was not administered DEN died due to lung hemorrhage. This can be explained by Kun MW *et al*., who discovered the other cause of Wistar rat’s lung problem was lung infection due to *A.Cantonensis.* ^35^ *M. Pulmonis* causes a different type of infection, as explained by the study of Chawla *et al.*, which showed gross and histopathological discoveries of severe congestion of the lungs with suppurative and necrotizing pneumonia. ^36^ Wang Y *et al.* also evaluated the induction effect of DEN in a rat model. They discovered that the induced rat had liver dysfunction and damage, characterized by diffuse lesions with extensive interstitial inflammatory cell infiltration, alveolar edema, and bleeding. Meanwhile, minor injuries were discovered in the spleen, kidney, large intestine, heart, and other organs. ^37^ Atmodjo *et al.* also noted the same discoveries for lung hemorrhage. ^33^

There are several limitations to this study; firstly, the unhealthy condition of the samples after ten weeks of administration of DEN can affect the number of samples. This led to the consideration of the decision of earlier termination. Secondly, the method of administering EGCG was unclear with the best effectiveness, oral EGCG, which is associated with poor absorption (<5% absorption rate). Therefore, the intraperitoneal injection method was used to administer EGCG. This study recommends further investigation of whether the administration of EGCG in the form of nanoparticles orally or parenterally can increase the absorption and bioavailability of EGCG in the intestine and plasma. ^12^^,^^13^

## Conclusion

The combination of low-dose Sorafenib with EGCG has a more potential anti-angiogenic effect on liver cancer by reducing VEGF levels compared to the single standard-dose Sorafenib. It also has similar effectiveness as single standard-dose Sorafenib in reducing MVD expression compared to the control group. Meanwhile, further study on the anti-angiogenic effect of low-dose sorafenib combined with EGCG is recommended to evaluate resistance and Toxicity.

## Data availability

### Underlying data

Zenodo: Underlying data for ‘Anti-angiogenic effect of the combination low-dose sorafenib and EGCG in HCC-induced Wistar rats.’ https://doi.org/10.5281/zenodo.6044890.

This project contains the following underlying data:

- VEGF and MVD raw data.xlsx (dataset)

### Reporting guidelines

Zenodo: ARRIVE checklist for ‘Anti-angiogenic effect of the combination low-dose sorafenib and EGCG in HCC-induced Wistar rats.’ https://doi.org/10.5281/zenodo.6044890.

Data are available under the terms of the Creative Commons Zero “No rights reserved” data waiver (CC0 4.0 Public domain dedication).

## Authors’ contributions

Conception and design: Andry Irawan, Erik Prabowo, Ignatius Riwanto

Administrative support: Andry Irawan, Erik Prabowo, Wahyuni Lukita Atmodjo

Provision of study materials or patients: Wahyuni Lukita Atmodjo

Collection and assembly of data: Andry Irawan

Data analysis and interpretation: Ignatius Riwanto, Wahyuni Lukita Atmodjo

Manuscript writing: All authors

Final approval of manuscript: All authors
