# Supplementary material for: Anti-angiogenic effect of the combination of low-dose sorafenib and EGCG in HCC-induced Wistar rats
Source: F1000Res. 2022 Dec 21;11:289. Originally published 2022 Mar 9. [Version 3] doi: 10.12688/f1000research.109142.3 (PMC9843086; doi:10.12688/f1000research.109142.3)
Supplement: Peer review history

# Peer review: Reviewer response for version 3

**DOI:** 10.5256/f1000research.142344.r158945

**Published:** 2023-01-16

**Authors:** Alessandro Granito

**Affiliations:** 1 Department of Medical and Surgical Sciences, Center for the Study and Treatment of Autoimmune Diseases of the Liver and Biliary System, IRCCS Azienda Ospedaliero-Universitaria di Bologna, University of Bologna, Bologna, Italy

In this study, the authors performed an animal study and double-blind, randomized controlled trials in 25 Wistar rats (7 weeks old) that were randomly divided into four groups, namely Sham (K), Control (O), a combination of low-dose Sorafenib and epigallo-3-catechin gallate group (X1), and standard-dose Sorafenib group (X2). All groups were injected with N-Nitrosodiethylamine 70 mg/kg body weight (BW) intraperitoneally for ten weeks, except the Sham group. After the development of hepatocellular carcinoma, X1 and X2 were treated for two weeks. They examined liver tissues and tumor masses for vascular endothelial growth factor (VEGF) level and microvascular density expression and found that there was a significant difference in the level of VEGF between group X1 (low dose Sorafenib + EGCG) and X2 (Standard dose Sorafenib). However, the differences in VEGF levels of group X1 and X2 compared to group O(Control) were significantly lower, with values p=0.000136 and p=0.019, respectively. They concluded that the combination of low-dose Sorafenib with epigallo-3-catechin gallate is superior in reducing the level of VEGF compared to standard-dose Sorafenib and is better than the control. Standard-dose Sorafenib and the combination of low-dose Sorafenib and epigallo-3-catechin gallate have similar effectivity in reducing the expression of microvascular density and could prevent resistance and lower toxicity effects.

 The study is of interest as it opens a new therapeutic approach based on the anti-angiogenic effect of low-dose sorafenib combined with EGCG.

 The clinical interest relies on the fact that most HCC patients have underlying liver cirrhosis that may limit the feasibility of antiangiogenic therapy.

 However, the authors should recall a similar approach has already been demonstrated with other low-dose-based treatments for hepatocellular carcinoma such as metronomic capecitabine. In fact, based on the rationale of the angiogenic effects of prolonged low-dose of therapy, it has been previously demonstrated that metronomic capecitabine is a safe and effective therapy for hepatocellular carcinoma patients, as previously reported ^1^^,^^2^.

Is the work clearly and accurately presented and does it cite the current literature?

Yes

If applicable, is the statistical analysis and its interpretation appropriate?

Yes

Are all the source data underlying the results available to ensure full reproducibility?

Yes

Is the study design appropriate and is the work technically sound?

Yes

Are the conclusions drawn adequately supported by the results?

Yes

Are sufficient details of methods and analysis provided to allow replication by others?

Yes

Reviewer Expertise:

Hepatocellular carcinoma diagnosis and treatments.

I confirm that I have read this submission and believe that I have an appropriate level of expertise to confirm that it is of an acceptable scientific standard, however I have significant reservations, as outlined above.

## References

[1] : Metronomic capecitabine as second-line treatment in hepatocellular carcinoma after sorafenib failure. *Dig Liver Dis* .2015;47(6) : 10.1016/j.dld.2015.03.010 518-22 10.1016/j.dld.2015.03.010 25861840

[2] : Metronomic Capecitabine in Patients With Hepatocellular Carcinoma Unresponsive to or Ineligible for Sorafenib Treatment: Report of Two Cases. *Hepatitis Monthly* .2013;13(9) : 10.5812/hepatmon.11721 10.5812/hepatmon.11721 PMC383051524282421

[3] : Systemic treatments for hepatocellular carcinoma: challenges and future perspectives. *Hepat Oncol* .2018;5(1) : 10.2217/hep-2017-0020 HEP01 10.2217/hep-2017-0020 30302192PMC6168042

# Peer review: Reviewer response for version 3

**DOI:** 10.5256/f1000research.142344.r158508

**Published:** 2023-01-05

**Authors:** Yefta Moenadjat

**Affiliations:** 1 Faculty of Medicine, Department of Surgery Cipto Mangunkusumo General Hospital, Universitas Indonesia, Jakarta, Indonesia

I approve the manuscript. No additional comments.

Is the work clearly and accurately presented and does it cite the current literature?

Partly

If applicable, is the statistical analysis and its interpretation appropriate?

Partly

Are all the source data underlying the results available to ensure full reproducibility?

Partly

Is the study design appropriate and is the work technically sound?

Partly

Are the conclusions drawn adequately supported by the results?

Partly

Are sufficient details of methods and analysis provided to allow replication by others?

Partly

Reviewer Expertise:

NA

I confirm that I have read this submission and believe that I have an appropriate level of expertise to confirm that it is of an acceptable scientific standard.

# Peer review: Reviewer response for version 2

**DOI:** 10.5256/f1000research.138994.r156194

**Published:** 2022-11-25

**Authors:** Yefta Moenadjat

**Affiliations:** 1 Faculty of Medicine, Department of Surgery Cipto Mangunkusumo General Hospital, Universitas Indonesia, Jakarta, Indonesia

Thanks for the revision. The revision has been shown compared to the previous one. However, some must be revised and completed (please find the comment in the sticky note).

Is the work clearly and accurately presented and does it cite the current literature?

Partly

If applicable, is the statistical analysis and its interpretation appropriate?

Partly

Are all the source data underlying the results available to ensure full reproducibility?

Partly

Is the study design appropriate and is the work technically sound?

Partly

Are the conclusions drawn adequately supported by the results?

Partly

Are sufficient details of methods and analysis provided to allow replication by others?

Partly

Reviewer Expertise:

NA

I confirm that I have read this submission and believe that I have an appropriate level of expertise to confirm that it is of an acceptable scientific standard, however I have significant reservations, as outlined above.

# Response

**Published:** 2022-12-17

**Authors:** Andry Irawan

**Affiliations:** Pelita Harapan University, Indonesia

Dear Dr Yefta Moenadjat,

 Thank you for allowing us to submit a revised draft of our manuscript. We have adjusted several texts and sentences based on your highlighted texts. We also attempted to register the experimental study; however, all animal study registries must be submitted before the research is done. We sincerely regret this matter, and our evaluation will be for the following animal study.

 We look forward to hearing from you regarding the submission, responses, questions, and comments you may have. Thank you.

 Sincerely,

 Andry Irawan

# Peer review: Reviewer response for version 1

**DOI:** 10.5256/f1000research.120607.r151185

**Published:** 2022-09-23

**Authors:** Andree Kurniawan

**Affiliations:** 1 Department of Internal Medicine, Faculty of Medicine, Pelita Harapan University, Tangerang, Indonesia

The authors have reported the basic science research regarding the the role of anti angiogenesis in HCC.

 My comments are:

 1. In the introduction may be added the recent update in HCC since there are emerging new data about anti angiogenesis and immunotherapy . may be added also the role of levantinib. Sorafenib nowadays is not a standard of care for advanced inoperable HCC. Atezo Bev was the standard of care.

 2. Should be added also in the introduction the background rationale for this research since there are several studies about it already.

 3. In the discussion should be added the implication for clinical practice the combination of experimental drugs. What is the implication for translation research from this data?

 4. Advice for further basic research should be added, related to the limitations of this study. 

 5. In the discussion do not repeat the results, however, compare the results with other studies - similar results or different results ?

Is the work clearly and accurately presented and does it cite the current literature?

Partly

If applicable, is the statistical analysis and its interpretation appropriate?

Partly

Are all the source data underlying the results available to ensure full reproducibility?

Yes

Is the study design appropriate and is the work technically sound?

Yes

Are the conclusions drawn adequately supported by the results?

Yes

Are sufficient details of methods and analysis provided to allow replication by others?

Yes

Reviewer Expertise:

HCC, epidemiology, cancer, hematology

I confirm that I have read this submission and believe that I have an appropriate level of expertise to confirm that it is of an acceptable scientific standard, however I have significant reservations, as outlined above.

# Response

**Published:** 2022-11-08

**Authors:** Andry Irawan

**Affiliations:** Pelita Harapan University, Indonesia

Dear Dr Andree Kurniawan,

 Thank you for allowing us to submit a revised draft of our manuscript titled *Anti-angiogenic effect of the combination low-dose sorafenib and EGCG in HCC-induced Wistar rats*. We appreciate the time and effort you have dedicated to providing your valuable feedback on our manuscript. We are grateful to the reviewer for their insightful comments on our paper. We have been able to incorporate changes to reflect most of the suggestions provided by the reviewer. We have highlighted the changes within the manuscript.

 Here is a point-by-point response to the reviewer's comments and concerns.

 1. In the introduction may be added the recent update in HCC since there are emerging new data about anti angiogenesis and immunotherapy may be added also the role of levantinib. Sorafenib nowadays is not a standard of care for advanced inoperable HCC. Atezo Bev was the standard of care.

**Author’s comment: **We have incorporated the additional statement in the second last paragraph of the introduction.

 2. Should be added also in the introduction the background rationale for this research since there are several studies about it already.

**Author’s comment: **The research rationale has been stated in the 3 ^rd^ paragraph of the introduction.

 3. In the discussion should be added the implication for clinical practice the combination of experimental drugs. What is the implication for translation research from this data?

**Author’s comment: **The combination of low-dose Sorafenib with EGCG has a more potential anti-angiogenic effect in liver cancer by reducing VEGF values compared to the single standard-dose Sorafenib. We can advance the research into human, but firstly we need to examine the toxicity effect in animal research.

 4. Advice for further basic research should be added, related to the limitations of this study. 

**Author’s comment: **The advice for further studies has been mentioned in the limitation section of the discussion.

 5. In the discussion do not repeat the results, however, compare the results with other studies - similar results or different results?

**Author’s comment: **The comparison with other studies has been incorporated in the 4 ^th^ paragraph of the discussion.

 We look forward to hearing from you in due time regarding the submission, responses, further questions, and comments you may have. Thank you.

 Sincerely,

 Andry Irawan

# Response

**Published:** 2022-12-17

**Authors:** Andry Irawan

**Affiliations:** Pelita Harapan University, Indonesia

Dear Dr. Yefta Moenadjat,

 Thank you for allowing us to submit a revised draft of our manuscript. We have adjusted several texts and sentences based on your highlighted texts. We also attempted to register the experimental study; however, all animal study registries must be submitted before the research is done. We sincerely regret this matter, and our evaluation will be for the following animal study.

 We look forward to hearing from you regarding the submission, responses, questions, and comments you may have. Thank you.

 Sincerely,

 Andry Irawan

# Peer review: Reviewer response for version 1

**DOI:** 10.5256/f1000research.120607.r141158

**Published:** 2022-06-22

**Authors:** Yefta Moenadjat

**Affiliations:** 1 Faculty of Medicine, Department of Surgery Cipto Mangunkusumo General Hospital, Universitas Indonesia, Jakarta, Indonesia

**1. The abstract**

- Abstract reflects the information of the content. The first statement in the background was not in line with the texts elsewhere in the content. In addition, the study was not dealing with the cost and benefit but the efficacy. Then why do the authors put an irrelevant statement here?
- The method section in the abstract comprises a brief description of the study design and PICO. Consider revising.
- In the results section of an abstract, it is better to provide the interpretation of the findings in the study rather than providing statistical analysis. In addition, it will be better to put an attribute (for instance, group I, II, II, and control) rather than X1, X2, confusing the readers.
- Results session: Please consider using English formatting correctly. English does not use a comma but a point for decimals.
- The abstract's conclusion differs from the conclusion written in the body and is not in line with the study's title and aim, which is focused on the anti-angiogenic effect. Therefore, consider being consistent with it.

**2. Introduction**

- The second paragraph:
  - Are the statement in the first three sentences referred to as the same reference as the fourth sentence?
  - There is an interplay of some factors, including VEGF inducing cancer - including HCC - and not solely VEGF. Consider elaborating a little bit regarding this. It does not matter if the authors solely focus on VEGF.
  - Consistency is essential in scientific publication. Therefore, consider using 'level' consistently instead of using a different term of 'concentration' or 'values' in this manuscript.
- The third paragraph:
  - Is surgery the most common procedure, or is it a primary or definitive procedure?
  - The author stated: "...while chemotherapy and targeted therapy are also used." The question is: Is it 'also used' or is it the protocol for the HCC?
- The fourth paragraph:
  - Instead of providing information from previous studies reporting improvement in the prognosis of HCC, the authors should propose the rationale for using this drug to suppress VEGF.
  - It is essential to answer the question of why use this drug.
  - Authors should provide information on why using the rat model. Particularly the HCC-induced model.
  - There is no information about using DEN in the background that presents the transparency of using DEN to induce HCC.
  - Consider providing such information about toxicity and carcinogenesis effect briefly. Not all readers know about this.
- The fifth paragraph:
  - The statement was not in line with the structure following the checklist. In addition, the link provided indicating the ARRIVE checklist is the same as provided in ARRIVE guidelines https://arriveguidelines.org/ but not specific for this study.

**3. Methods**

- Describe systematically and sequentially:
  - Study design, division of groups in randomization (including randomization process, random? who did the randomization? what was the instrument used for this purpose?)
  - Preparation of animals and pre-intervention treatment,
  - Intervention detail in each group,
  - Preparation of materials/materials.
- Next, the treatment consists of:
  - The carcinogenesis induction process. Who judges the successful induction, and how did it assess, showing that the intervention was successful? How long does it take to determine that the induction process was successful?
- Describe further detail of tumor-induced DEN characteristics that are similar to an HCC.
- This section does not explain when Sorafenib and ECGC are given. Either immediately or wait until the HCC induction procedure has been successful. Although, it was explained that the specimens were taken ten weeks after administering these two drugs.
- The authors did not describe the specific area of the taken specimen but ‘liver tissue’. Was it taken from a tumor mass?
- Describe further detail of method in systematic order.

**4. Results**. Use the same terminology that commented in the introduction and method. Consider providing the findings, but not the interpretation.

**5. Discussion**. Use the same terminology that commented in the introduction and method.

**6. Conclusion**. Use the same terminology that commented in the introduction and method.

Is the work clearly and accurately presented and does it cite the current literature?

Partly

If applicable, is the statistical analysis and its interpretation appropriate?

Partly

Are all the source data underlying the results available to ensure full reproducibility?

Partly

Is the study design appropriate and is the work technically sound?

Partly

Are the conclusions drawn adequately supported by the results?

Partly

Are sufficient details of methods and analysis provided to allow replication by others?

Partly

Reviewer Expertise:

NA

I confirm that I have read this submission and believe that I have an appropriate level of expertise to confirm that it is of an acceptable scientific standard, however I have significant reservations, as outlined above.

# Response

**Published:** 2022-09-22

**Authors:** Andry Irawan

**Affiliations:** Pelita Harapan University, Indonesia

Dear Dr Yefta Moenadjat,

 Thank you for allowing us to submit a revised draft of our manuscript titled *Anti-angiogenic effect of the combination low-dose sorafenib and EGCG in HCC-induced Wistar rats*. We appreciate the time and effort you have dedicated to providing your valuable feedback on our manuscript. We are grateful to the reviewer for their insightful comments on our paper. We have been able to incorporate changes to reflect most of the suggestions provided by the reviewer. We have highlighted the changes within the manuscript.

 Here is a point-by-point response to the reviewer's comments and concerns.

**1. THE ABSTRACT**

- Abstract reflects the information of the content. The first statement in the background was not in line with the texts elsewhere in the content. In addition, the study was not dealing with the cost and benefit but the efficacy. Then why do the authors put an irrelevant statement here?
- The method section in the abstract comprises a brief description of the study design and PICO. Consider revising.
- In the results section of an abstract, it is better to provide the interpretation of the findings in the study rather than providing statistical analysis. In addition, it will be better to put an attribute (for instance, group I, II, II, and control) rather than X1, X2, confusing the readers.
- Results session: Please consider using English formatting correctly. English does not use a comma but a point for decimals.
- The abstract's conclusion differs from the conclusion written in the body and is not in line with the study's title and aim, which is focused on the anti-angiogenic effect. Therefore, consider being consistent with it.

**Author’s comment:** *Thank you for your concern. We have revised the abstract section into a more relevant statement.*

**2. INTRODUCTION**

 The second paragraph: 

- Are the statement in the first three sentences referred to as the same reference as the fourth sentence?

**Author’s comment:** Yes, they referred to the same reference.

- There is an interplay of some factors, including VEGF inducing cancer - including HCC - and not solely VEGF. Consider elaborating a little bit regarding this. It does not matter if the authors solely focus on VEGF
- Consistency is essential in scientific publication. Therefore, consider using 'level' consistently instead of using a different term of 'concentration' or 'values' in this manuscript.

**Author’s comment:** *Thank you for your positive feedback. We have elaborated the interplay factors and revised the ‘concentration’ or ‘values’ into ‘level’ terms.*

 The third paragraph: 

- Is surgery the most common procedure, or is it a primary or definitive procedure?

**Author’s comment:** Yes, surgery is the definitive treatment for HCC if it is operable

- The author stated: "...while chemotherapy and targeted therapy are also used." The question is: Is it 'also used' or is it the protocol for the HCC?

**Author’s comment: **Yes, it is the protocol for unresectable HCC

 The fourth paragraph: 

- Instead of providing information from previous studies reporting improvement in the prognosis of HCC, the authors should propose the rationale for using this drug to suppress VEGF.
- It is essential to answer the question of why use this drug.
- Authors should provide information on why using the rat model. Particularly the HCC-induced model.
- There is no information about using DEN in the background that presents the transparency of using DEN to induce HCC.
- Consider providing such information about toxicity and carcinogenesis effect briefly. Not all readers know about this.

**Author’s comment: **Thank you for your considerate question. We have incorporated all the questions stated above within the manuscript.

 The fifth paragraph: 

- The statement was not in line with the structure following the checklist. In addition, the link provided indicating the ARRIVE checklist is the same as provided in ARRIVE guidelines https://arriveguidelines.org/ but not specific for this study.

**Author’s comment: **The ARRIVE checklist is available in the reporting guideline section. Zenodo: ARRIVE checklist for ‘Anti-angiogenic effect of the combination low-dose sorafenib and EGCG in HCC-induced Wistar rats.’ https://doi.org/10.5281/zenodo.6044890.

**3. METHODS**

 Describe systematically and sequentially: 

- Study design, division of groups in randomization (including randomization process, random? who did the randomization? what was the instrument used for this purpose?)

**Author’s comment: **This study was a double-blind, randomized control trial. A laboratory analyst performed randomization into four groups.

- Preparation of animals and pre-intervention treatment,
- Intervention detail in each group,
- Preparation of materials/materials.

**Author’s comment: **Thank you for your insightful comments. We have incorporated the additional statement in the methods section.

 Next, the treatment consists of: 

- The carcinogenesis induction process. Who judges the successful induction, and how did it assess, showing that the intervention was successful? How long does it take to determine that the induction process was successful?

**Author’s comment: **An anatomical pathologist from Dr Mintoharjo Naval Hospital determined the successful induction under the microscope and required ten weeks to confirm the accomplishment of the induction.

- Describe further detail of tumor-induced DEN characteristics that are similar to an HCC.

**Author’s comment**: We have elaborated on the similarity of the tumor-induced DEN and HCC characteristics.

- This section does not explain when Sorafenib and ECGC are given. Either immediately or wait until the HCC induction procedure has been successful. Although, it was explained that the specimens were taken ten weeks after administering these two drugs.

**Author’s comment: **Sorafenib and ECGC were given until the HCC induction procedure was successful by the result from the anatomical pathologist successfully succeeded.

- The authors did not describe the specific area of the taken specimen but ‘liver tissue’. Was it taken from a tumor mass?

**Author’s comment: **Yes, we took the whole liver tissue-contained tumor masses

- Describe further detail of method in systematic order.

**Author’s comment:** We have amended the method section more systematically.

 We look forward to hearing from you in due time regarding the submission, responses, further questions, and comments you may have. Thank you.

 Sincerely,

 Andry Irawan
